# TNFSF15 inhibits progression of diabetic retinopathy by blocking pyroptosis via interacting with GSDME

**DOI:** 10.1038/s41419-024-06499-8

**Published:** 2024-02-08

**Authors:** Hongkun Zhao, Yating Dai, Yunqin Li, Juanjuan Li, Hua Li

**Affiliations:** 1 Key Laboratory of Yunnan Province, Yunnan Eye Institute, Affiliated Hospital of Yunnan University, Yunnan University, Kunming, Yunnan China; 2 Department of Pathology and Pathophysiology, Faculty of Basic Medical Sciences, Kunming Medical University, Kunming, China

**Keywords:** Cell death, Proteins

## Abstract

Diabetic retinopathy is a common microvascular complication of diabetes and a leading cause of blindness. Pyroptosis has emerged as a mechanism of cell death involved in diabetic retinopathy pathology. This study explored the role of GSDME-mediated pyroptosis and its regulation by TNFSF15 in diabetic retinopathy. We found GSDME was upregulated in the progression of diabetic retinopathy. High glucose promoted GSDME-induced pyroptosis in retinal endothelial cells and retinal pigment epithelial cells, attributed to the activation of caspase-3 which cleaves GSDME to generate the pyroptosis-executing N-terminal fragment. TNFSF15 was identified as a binding partner and inhibitor of GSDME-mediated pyroptosis. TNFSF15 expression was increased by high glucose but suppressed by the caspase-3 activator Raptinal. Moreover, TNFSF15 protein inhibited high glucose- and Raptinal-induced pyroptosis by interacting with GSDME in retinal cells. Collectively, our results demonstrate TNFSF15 inhibits diabetic retinopathy progression by blocking GSDME-dependent pyroptosis of retinal cells, suggesting the TNFSF15-GSDME interaction as a promising therapeutic target for diabetic retinopathy.

## Introduction

Diabetic retinopathy (DR) is a severe microvascular complication of diabetes and a leading cause of vision loss globally. It is characterized by progressive damage to the retinal microvasculature [1]. The early stage of DR is called nonproliferative DR (NPDR), which manifests as microaneurysms, hemorrhages, and vascular leakage due to the breakdown of the blood-retinal barrier. Without timely treatment, NPDR can advance to proliferative DR (PDR), characterized by pathologic neovascularization and fibrosis, which may lead to severe vision loss due to vitreous hemorrhage and retinal detachment [2, 3]. Despite the therapeutic efficacy of anti-VEGF agents for DR, limitations exist including the need for frequent intravitreal injections, prolonged treatment cycles, transient efficacy, and poor response in some patients [4–6]. The risk of vision loss persists in DR patients.

Pyroptosis is a lytic and inflammatory form of programmed cell death orchestrated by the cleavage of the pore-forming protein gasdermin family proteins [7–9]. Upon pathogenic stimuli, gasdermins are cleaved and oligomerize to form pores on the plasma membrane, leading to cell lysis and release of proinflammatory contents [10]. Emerging evidence implicates pyroptosis of retinal pericytes and endothelial cells in DR pathogenesis [11, 12]. Under hyperglycemic conditions, NLRP3 inflammasomes in retinal pericytes are activated to cleave gasdermin D (GSDMD), executing pyroptosis [13]. Pyroptotic pericytes release inflammatory cytokines IL-1β and IL-18, which impair endothelial cells and capillaries [13]. In retinal endothelial cells, high glucose stimulates TLR4 and NF-κB signaling to activate caspase-3, triggering endothelial pyroptosis [14]. Pyroptotic retinal endothelial cells propagate inflammation and compromise blood-retinal barrier integrity via P2X7/NLRP3 signaling pathway, resulting in DR [15]. gasdermin E (GSDME), another gasdermin family mediator of cell death and inflammation, is cleaved by caspase-3 into functional N-terminal pore-forming fragments [16]. Bioinformatic analysis revealed GSDME upregulation in diabetic retinas, implicating its pyroptotic role in DR [6]. However, the precise functions and mechanisms of GSDME in DR pathogenesis remain elusive.

Vascular endothelial growth factor (VEGF) has been implicated in the pathogenesis of DR [17], its upregulation in the retina in diabetes contributes to increased vascular permeability, edema, and pathological neovascularization associated with DR [18]. On the one hand, Conbercept is a promising anti-VEGF fusion protein developed in China that has shown efficacy in improving vision and reducing retinal thickness in DR patients [19]. Multiple clinical trials have demonstrated that intravitreal injections of conbercept significantly improve visual acuity and reduce central retinal thickness compared to placebo or contrast agents in DR patients [20, 21]. On the other hand, Tumor necrosis factor superfamily member 15 (TNFSF15) is an inhibitor of VEGF in DR [22, 23]. Under hyperglycemic conditions, TNFSF15 significantly inhibits VEGF expression in the retina through dual mechanisms [23]. Additionally, TNFSF15 directly sequesters VEGF by binding, thereby attenuating its interaction with VEGFR and downstream bioactivities [24]. As such, TNFSF15 negatively regulates VEGF in retinal pathology via both indirect and direct mechanisms. Recent studies demonstrate TNFSF15 can mitigate VEGF-induced endothelial hyperpermeability and pathologic neovascularization in the retina, conferring vascular protection [23, 24]. Its anti-angiogenic and vascular stabilizing effects highlight TNFSF15 as a promising therapeutic target for DR.

We found that under hyperglycemic conditions, GSDME is activated in retinal cells, inducing pyroptosis and promoting DR progression. TNFSF15 can interact with GSDME, thereby inhibiting GSDME-triggered pyroptosis. Functional experiments demonstrate TNFSF15 significantly suppresses GSDME-induced retinal cell pyroptosis. Additionally, the anti-VEGF agent Conbercept inhibits high glucose-induced retinal cell pyroptosis, while TNFSF15 overexpression also markedly suppresses hyperglycemia-induced pyroptosis of retinal capillary endothelial cells. This study uncovers a novel molecular mechanism whereby TNFSF15 protects against retinal pathogenesis by inhibiting GSDME-mediated pyroptosis. It provides a rationale for developing novel DR therapies targeting the TNFSF15-GSDME axis.

## Materials and methods

### Cell culture

The ARPE-19 and HRMECs cell line were purchased from ATCC. ARPE-19 cells were cultured in DMEM/F12 medium supplemented with 10% fetal bovine serum and 1% penicillin–streptomycin. HRMECs cells were cultured in complete medium purchased from ATCC (STR authenticated). Then cells were maintained at 37 °C in a humidified 5% CO_2_ incubator. When the confluence reached 80–90%, cells were digested with 0.25% trypsin-EDTA for passaging. Prior to experiments, ARPE-19 cells and HRMECs cells were tested for mycoplasma contamination to ensure there was no infection. Experiments were conducted after the cells adhered to the plates.

### Chemicals and reagents

The following reagents were used: STZ (S0130, Sigma-Aldrich), Raptinal (Rap) (SML1745, Sigma-Aldrich), TNFSF15 protein (Cell Signaling Technology, #11946), Conbercept (Kanghong Pharmaceuticals), Glucose (G7021, Sigma-Aldrich), 4,6-diamidino-2-phenylindole (DAPI) (D9542, Sigma-Aldrich), BCA protein assay kit (23250, Thermo 239 Scientific), lipofectamine 3000 (2274248, Invitrogen), bovine serum albumin (BSA) (New England BioLabs), enhanced chemiluminescence (ECL) detection reagents (32106, Thermo Fisher Scientific), polyethylenimine (PEI) (Polysciences), paraformaldehyde (PFA) (158127, Sigma-Aldrich), poly-lysine (P4707, Sigma-Aldrich), protease inhibitors cocktail tablet (4693132001, Roche, Basel, Switzerland), restriction enzymes (New England BioLabs), RIPA buffer (#9806, Cell Signaling Technology), DTT (ST043, Beyotime), sodium deoxycholate (Doc.NA) (D6750, Sigma-Aldrich), IGEPAL CA-630 (I3021, Sigma-Aldrich), PMSF (A100754, Diamond), Triton X-100 (T8787, Sigma-Aldrich), 2-mercaptoethanol (M6230, MACKLIN) pEASY®-Basic Seamless Cloning and Assembly Kit (CU201, TransGen Biotech), Trans2K® Plus II DNA Markers (BM121, TransGen Biotech), and Page Ruler^TM^ Prestained Protein Ladder (26616, Thermo Scientific).

Horseradish peroxidase-conjugated secondary antibodies were purchased from Bio-Rad, USA. Mouse Anti-Rabbit (93702) /Mouse (58802) IgG (Light-Chain Specific) were purchased from Cell Signaling Technology. Primary antibodies used in this study are listed in the Table 1.

**Table 1:** Primary antibodies used in this study.

Primary antibody		Dilution ratio			Supplier
		IHC	*is*PLA	WB	
ACTB	Mouse			1:10,000	Abcam, ab8226
GSDME	Rabbit	1:100	1:100	1:2000	Abcam, 215191; Proteintech, 13075-1-AP
Flag	Mouse	1:400	1:400	1:1000	Sigma-Aldrich, F7425
HA	Rabbit	1:400	1:400	1:1000	Cell Signaling Technology, 3724
Caspase −3	Rabbit	1:200	1:200	1:1000	Cell Signaling Technology, 9662
Cleaved Caspase-3	Rabbit	1:200	1:200	1:1000	Cell Signaling Technology, 9661

### Construction of Type 1 and Type 2 diabetic rat models

Adult male Sprague-Dawley rats (200–250 g, male, age 4~6w) were obtained from the Experimental Animal Center of Yunnan University and housed under a 12 h light/dark cycle with free access to food and water. The rats were allowed to acclimatize for 1 week prior to experiments. Every effort was made to minimize animal suffering. At the end of the experiments, rats were euthanized by CO_2_ inhalation. All procedures were performed in accordance with the Regulations for the Administration of Affairs Concerning Experimental Animals approved by the State Council of China. In determining the sample size, taking into account ethical concerns, resource constraints, and the number of samples conforming to the *t*-test statistical analysis, we selected total 200 rats for inducing type 1 and 2 diabetic rats. Without the use of statistical analysis, six rats in each group met the requirements of the experimental sample size.

To induce type 1 diabetes, healthy male Sprague-Dawley (SD) rats (220–250 g, male, age 4~6w) were fasted overnight before receiving a single dose of freshly prepared streptozotocin (STZ; 60 mg/kg body weight) by intraperitoneal injection. STZ was dissolved in citric acid buffer (0.1 M, pH 4.2) prior to injection. At 72 h post-injection, tail vein blood glucose was analyzed and rats with glucose levels ≥16.7 mmol/L were considered successfully diabetic. To generate a type 2 diabetic model, male SD rats (190–210 g) were fed a high-fat diet for 4 weeks followed by a low dose STZ injection (30 mg/kg body weight, intraperitoneal). Blood glucose was monitored at 72 h post injection and weekly thereafter. Rats exhibiting random blood glucose between 16.7–20 mmol/L were considered to have developed type 2 diabetes. At 9 and 13 weeks following establishment of diabetes, eyeballs were enucleated and retinas isolated for subsequent histological, immunohistochemical and immunofluorescence analyses. Inclusion criteria for subsequent animal experiments: successful establishment of diabetes model. Exclusion criteria: No disease other than diabetes was present. The experimental animals were randomly assigned to the experimental and control groups. In each of the subsequent experiments, the number of diabetic rats was 6. Each experiment was repeated three times. The animal experiments were conducted in a no blind way.

### Transcriptome sequencing (RNA-seq)

RNA-seq data was published on Sequence Read Archive (SRA) database (BIOproject number: PRJNA1059505). RNA-seq was performed on HRMECs cells to analyze transcriptomic changes. HRMECs cells were divided into normal control (NC, *n* = 2), Raptinal-treated (NCR, *n* = 2), high glucose (HG, *n* = 2) and Raptinal-treated+HG (HGR, *n* = 2)groups. Total RNA was extracted from cultured HRMECs cells using TRIzol reagent. The subsequent library construction and sequencing steps were the same as described above. Clean reads were aligned to the human reference genome. Gene expression levels were quantified and compared between NC and NCR groups to identify differentially expressed genes after Raptinal treatment in HRMECs cells. RNA sequencing (RNA-seq) was performed to analyze the transcriptome profiles of retinal tissues. Total RNA was extracted using TRIzol reagent. RNA purity was checked by NanoPhotometer spectrophotometer and integrity was assessed using the RNA Nano 6000 Assay Kit on the Bioanalyzer 2100 system. Sequencing libraries were generated using NEBNext Ultra RNA Library Prep Kit for Illumina following manufacturer’s recommendations. Briefly, mRNA was purified from total RNA using poly-T oligo-attached magnetic beads and fragmented. First strand cDNA was synthesized using random hexamer primer and M-MuLV Reverse Transcriptase. Second strand cDNA synthesis was subsequently performed using DNA Polymerase I and RNase H. After adenylation of 3′ ends of DNA fragments, NEBNext Adaptor was ligated for hybridization. PCR was then carried out to enrich cDNA fragments with adaptor molecules on both ends and amplify the amount of DNA in the library. The Agilent Bioanalyzer 2100 system was used to assess library quality. The libraries were sequenced on an Illumina HiSeq platform to generate 150 bp/125 bp paired-end reads. Raw reads were processed and filtered using Cutadapt and Perl scripts. Clean reads were mapped to the reference genome using STAR. Gene expression levels were quantified by RSEM. Differential expression analysis was performed using the DESeq2 R package.

### Transmission electron microscopy

ARPE-19 cells and HRMECs were divided into normal control (NC) and high glucose (HG, 50 mM glucose) groups. ARPE-19 cells were then treated with Raptinal (2 μM). Afterwards, cells were collected and fixed with 2.5% glutaraldehyde overnight at 4 °C for transmission electron microscopy analysis.

After fixation, cells were rinsed with PBS and post-fixed in 1% osmium tetroxide for 1–2 h. Then the samples were dehydrated using graded ethanol, infiltrated with propylene oxide-Araldite resin, and embedded. Ultrathin sections (60–80 nm) were cut and mounted on copper grids. The sections were stained with uranyl acetate and lead citrate, then examined under a Hitachi H-7650 transmission electron microscope to observe apoptotic changes. Images were captured with an AMT 2k CCD camera. Each experiment was repeated three times.

### IF/IHC staining

Retinal paraffin sections (5 μm thick) were deparaffinized, rehydrated, and then subjected to antigen retrieval by microwave heating in citrate buffer. After cooling, the sections were washed with PBS, permeabilized with 0.2% Triton X-100 and blocked with 5% BSA. Then the sections were incubated with primary antibody in Table 1 at 4 °C overnight. On the next day, after PBS washing, the sections were incubated with secondary antibody for 1 h at room temperature. IF staining cell nuclei were counterstained with DAPI. The sections were imaged under a microscope to visualize GSDME/TNFSF15 expression. Each experiment was repeated three times.

### Western blotting assay

ARPE-19 cells and HRMECs cells were lysed with RIPA buffer containing protease inhibitors. Protein concentrations were determined using a BCA assay. Equal amounts of protein samples were separated by SDS-PAGE and transferred to PVDF membranes. After blocking with 5% non-fat milk, the membranes were incubated with primary antibodies at 4 °C overnight. Then the membranes were incubated with HRP-conjugated secondary antibodies (1:5000) for 1 h at room temperature. Protein bands were visualized using ECL reagent and imaged on a Gel Documentation System. β-actin was used as a loading control. Each experiment was repeated three times.

### in situ PLA assay

The *is*PLA experiment in 293T cell was conducted according to the instructions of the Duolink In Situ Detection Reagents Red kit (Sigma-Aldrich). First, cells were collected, followed by permeabilization and blocking. Next, primary antibody incubation was performed by diluting Flag and HA antibodies at 1:1000 and incubating overnight at 4 °C. Subsequently, secondary antibody incubation was carried out by incubating at 37 °C for 1–2 h, followed by ligation at 37 °C for 30 min. After that, amplification was performed at 37 °C for 3 h, then detection was allowed to proceed at room temperature for 1 h. DAPI nuclear staining was then done at room temperature for 5 min before mounting. Finally, imaging was performed using a microscope or confocal laser scanning microscope to visualize the results. Each experiment was repeated three times.

### Co-Immunoprecipitation

293T cells were collected 48 h after transfection. The cell culture medium was removed as much as possible using a pipette, and the cells were washed twice with pre-cooled PBS. An appropriate amount of cell lysis buffer was added, containing 50 mM Tris-HCl, pH 7.4, 150 mM sodium chloride, 5% glycerol, 1% IGEPAL CA-630, and 1 × cocktail protease inhibitor. The cells were placed on a rocker in a 4 °C cold room and rocked gently for 30 min for complete lysis. The cells were then collected into 1.5 mL centrifuge tubes and further disrupted by ultrasonication with settings of 30% ultrasonic energy, 1 min total ultrasonic time, 1 s ultrasonic time, and 1 s interval time. After ultrasonication, the samples were centrifuged at 12000 rpm for 20 min at 4 °C. The supernatant was collected into new 1.5 mL centrifuge tubes. Fifty microliters of protein G was added to the cell lysis buffer and rotated at low speed in the 4 °C cold room for 2 h to remove nonspecific contaminants. The cell lysate was collected at 4 °C and centrifuged at 1000 rpm for 5 min. The protein A/protein G was discarded and the supernatant was collected. One hundred microliters of the protein lysate was taken as Input.

#### Antibody incubation

The cell lysates were evenly divided into two groups. One group was incubated with anti-IgG antibody as a control, and the other group was incubated with 3 μL of anti-Flag or anti-HA antibody. The samples were incubated with primary antibody overnight at 4 °C with rotation.

#### Protein G incubation

On the second day, the cell lysates were taken out of the cold room and 20–50 μL of protein G was added. The samples were incubated at 4 °C with low-speed rotation for 5 h.

#### Washing protein G

After protein G incubation, the samples were centrifuged at 4 °C and 1000 rpm for 5 min. The supernatant was discarded and the beads were collected. 1 mL of lysis buffer was added and the sample rotated at 4 °C for 5 min. This wash step was repeated 6 times. Protein loading buffer was then added and the protein samples were heated at 100 °C for 10 min in a metal bath. The samples were stored at −20 °C for subsequent immunoblotting experiments. Each experiment was repeated three times.

### LDH realese assay

The LDH release assay is used to quantify cytotoxicity by measuring the activity of LDH released from damaged cells. First, cells are cultured in 96-well plates and exposed to experimental treatments or controls for a defined period of time. After treatment, the 96-well plate is centrifuged to pellet cells, and an aliquot of the supernatant from each well is transferred to a new 96-well enzymatic assay plate. The enzymatic assay plate contains freshly prepared LDH reaction mixture consisting of lactate, NAD+, diaphorase, and a tetrazolium salt. LDH in the transfered supernatant catalyzes the conversion of lactate to pyruvate via NAD+ reduction to NADH. The NADH then reduces the tetrazolium salt to a colored formazan product that absorbs at 490-520 nm. The absorbance of the formazan is proportional to LDH activity in the supernatant and is quantified using a microplate reader.Higher absorbance indicates greater LDH release due to cell damage. LDH release is calculated relative to maximal LDH release from fully lysed cells. Each experiment was repeated three times.

### Molecular docking

The AlphaFold2 program was used to model GSDME and TNFSF15, thereby obtaining complete three-dimensional structure models of GSDME and TNFSF15 proteins that can be used in subsequent molecular recognition studies. To study the binding regions and interaction modes between GSDME and TNFSF15 proteins, this study used the professional protein–protein and protein-DNA/RNA docking HDCOK program for docking, selecting the structure with the best docking score as the standard result for subsequent interaction analysis. The docking score is based on the ITScorePP or ITScorePR iterative scoring function. The more negative the docking score, the greater the binding probability and stronger the interaction between the binding models, but without experimental data correction, this score cannot be directly regarded as the true affinity value between two molecules.

Taking into account that the docking scores of protein–protein/RNA/DNA complexes in the PDB usually range from −200 or better, we have defined a docking score-dependent confidence score based on experience to represent the binding possibility of two molecules as follows: Confidence_score = 1.0/[1.0 + e0.02*(Docking_Score+150)]. Roughly speaking, when the confidence score is higher than 0.7, there is a high probability that the two molecules will bind; when the confidence score is between 0.5 and 0.7, there is a possibility that these two molecules will bind; when the confidence score is below 0.5, there is a low probability that these two molecules will bind. This study further analyzed the spatial structure binding sites and interaction modes between GSDME and TNFSF15 proteins, analyzed the hydrogen bond interactions and salt bridge interactions formed between GSDME and TNFSF15 proteins, as well as the specific amino acids involved in forming hydrogen bonds between proteins.

### Statistical analysis

Our experimental data were summarized as the mean ± standard error or mean ± deviation and statistically analyzed using a t-test and ANOVA. Graphpad 7 was used for statistical analysis. A *P* value of less than 0.05 was considered statistically significant. For every figure, statistical tests were justified as appropriate.

## Results

### GSDME is upregulated in the progression of DR

**Fig. 1:**
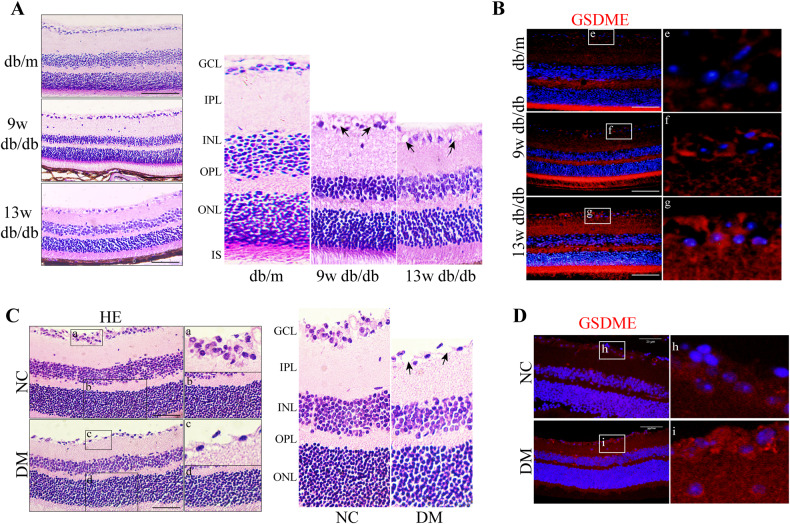
GSDME is upregulated in DR. **A** H&E staining of retinal sections from db/m and db/db rats at different ages in a type 2 diabetic rat model. Scale bars, 100 μm. Black arrows: vacuoles. **B** Immunofluorescence staining for GSDME protein in retinal sections from db/m and db/db rats at different ages in a type 2 diabetic rat model. Scale bars, 20 μm. **C** H&E staining of retinal sections from NC and DM rats in a type 1 diabetic rat model. Scale bars, 100 μm. Black arrows: vacuoles. **D** Immunofluorescence staining for GSDME protein in retinal sections from NC and DM rats in a type 1 diabetic rat model. Scale bars, 20 μm.

Analysis of potential pyroptosis-related genes involved in the occurrence of DR in GEO databases showed GSDME was a significant candidate gene [22]. To investigate the expression of GSDME in DR, type 1 (DM) and type 2 (db/db) diabetic rat models were generated – the former by single streptozotocin (STZ) injection and the latter by high-fat feeding plus low-dose STZ. By 9 weeks post-induction, both models exhibited retinal pathological alterations compared to controls, including disorganization, thinning from ganglion cell layer (GCL) to outer nuclear layer (ONL), loose layer arrangement, disrupted inner limiting membrane (ILM), and increased vacuolization (Fig. 1A, B). These changes worsened by 13 weeks in type 2 diabetic rats (Fig. 1B). Overall, the rapid manifestation of retinal abnormalities by 9 weeks demonstrates the utility of these models for elucidating early mechanisms of DR.

Immunofluorescence staining of GSDME revealed significant upregulation of the pyroptosis mediator protein GSDME in retinal tissues of both type 1 and type 2 diabetic rat models compared to non-diabetic control rats (Fig. 1C, D). These data suggest that GSDME is upregulated in the progress of DR.

### High glucose promotes GSDME induced pyroptosis in retinal cells

**Fig. 2:**
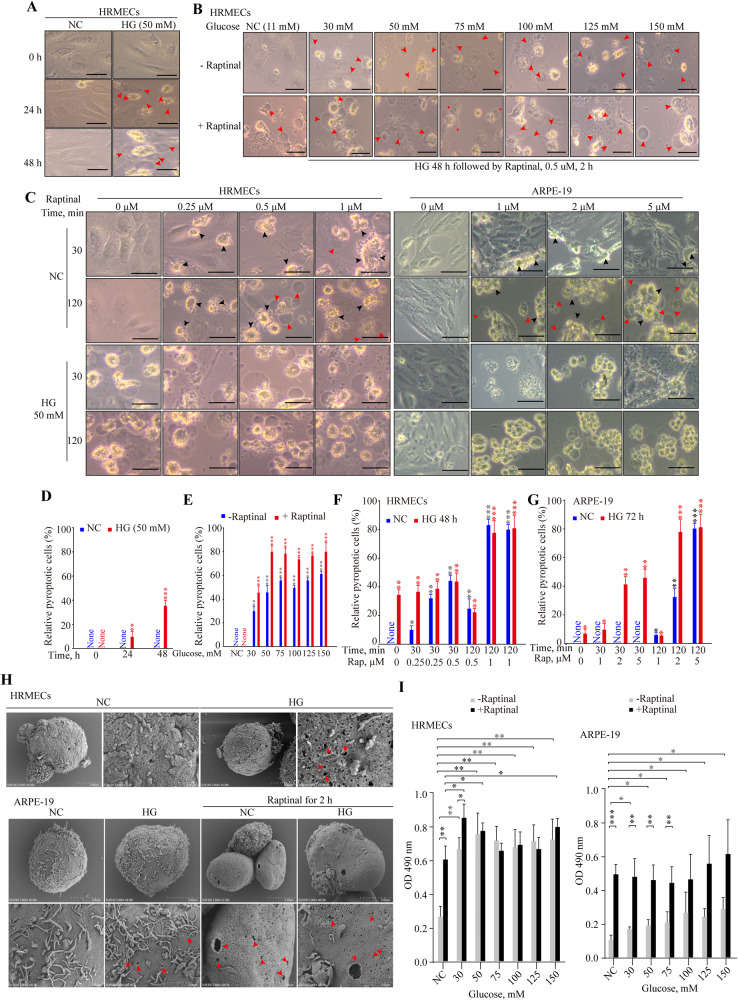
High glucose induces pyroptosis in retinal cells. **A**–**C** Morphology of ARPE-19 and HRMECs cells treated with different concentrations of glucose and/or Raptinal at different concentrations observed under optical microscopy. Black arrows: other forms of cell death such as apoptosis. Red arrows: swollen balloon-like pyroptotic cells. Scale bars, 50 μm. **D**–**G** Relative quantitation of pyroptotic cells among different groups. *_*P*_ < 0.01, **_*P*_ < 0.001, ***_*P*_ < 0.0001. **H** ARPE-19 and HRMECs cells were divided into NC, NC + Raptinal (1 μM), HG (50 mM) and HG (50 mM) + Raptinal (1 μM) groups. After 48 h of high glucose treatment, Raptinal was added for 1 h before cells were collected for scanning electron microscopy. **I** LDH release assay of ARPE-19 and HRMECs cells treated with different concentrations of glucose for 48 h. *P < 0.01, **P < 0.001, ***P < 0.0001.

To elucidate the role of GSDME in DR, human retinal microvascular endothelial cells (HRMECs) and human retinal pigment epithelial cells (ARPE-19) were treated with different high concentrations of glucose to mimic the continuous diabetic milieu in vivo. High glucose induced pyroptosis phenotype in both HRMECs and ARPE-19 in a concentration- and time-dependent manner, as evidenced by numerous balloon-like bubbles emerging on cell membranes (Fig. 2A–C). Accordingly, supplementing high glucose medium with Raptinal, a caspase-3 activator, sensitized HRMECs and ARPE-19 cells to high glucose, resulting in rapid and substantial pyroptosis (Fig. 2B–G). Numerous pores formed by GSDME N-terminal fragments on membranes of cells treated with high glucose and Raptinal under electron microscopy, compared to controls (Fig. 2H). These cells also exhibited significantly elevated lactate dehydrogenase release (Fig. 2I). Taken together, these experimental data demonstrate that high glucose induces and prmotes GSDME-mediated pyroptosis in retinal cells.

### High glucose activates caspase-3 to cleave GSDME

**Fig. 3:**
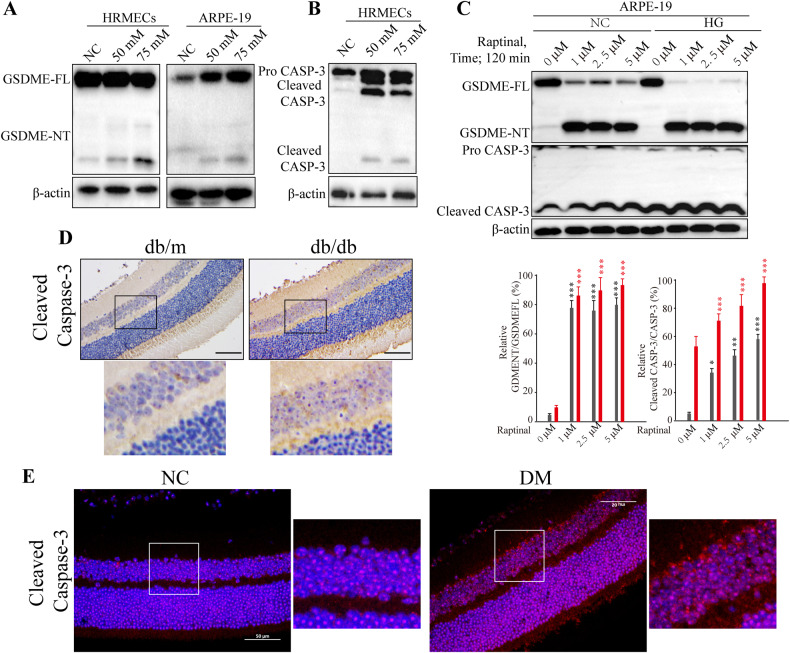
High glucose activates intracellular caspase-3 to cleave GSDME. **A**–**C** Western blot analysis of GSDME and caspase-3 protein expression changes in ARPE-19 and HRMECs cells after pretreatment with different glucose concentrations, and combined treatment with different concentrations of Raptinal for 2 h. *P < 0.01, **P < 0.001, ***P < 0.0001. **D**, **E** IHC (scale bars, 200 μm) and IF (scale bars, 100 μm) staining images of cleaved-caspase-3 in retinal tissues from NC and DM rats in type 1 and type 2 diabetic rat models.

To elucidate the mechanism underlying high glucose-induced pyroptosis in retinal cells, we analyzed the expression of GSDME and caspase-3. As anticipated, high glucose markedly upregulated the proportion of GSDME NT expression in both HRMECs and ARPE-19 cells (Fig. 3A, B), attributable to the conversion of pro-caspase-3 into active cleaved-caspase-3, which cleaves GSDME to generate the NT fragment. This effect was further enhanced upon treatment with the caspase-3 activator Raptinal (Fig. 3C–E). In agreement with the in vitro findings, caspase-3 expression was considerably elevated in retinal tissues of type 1 and 2 diabetic rats compared to control counterparts. Taken together, these results demonstrate that the high glucose microenvironment promotes caspase-3 expression and activation in retinal cells in vivo and in vitro, consequently increasing GSDME cleavage and eliciting pyroptosis.

### TNFSF15 interacts with GSDME

**Fig. 4:**
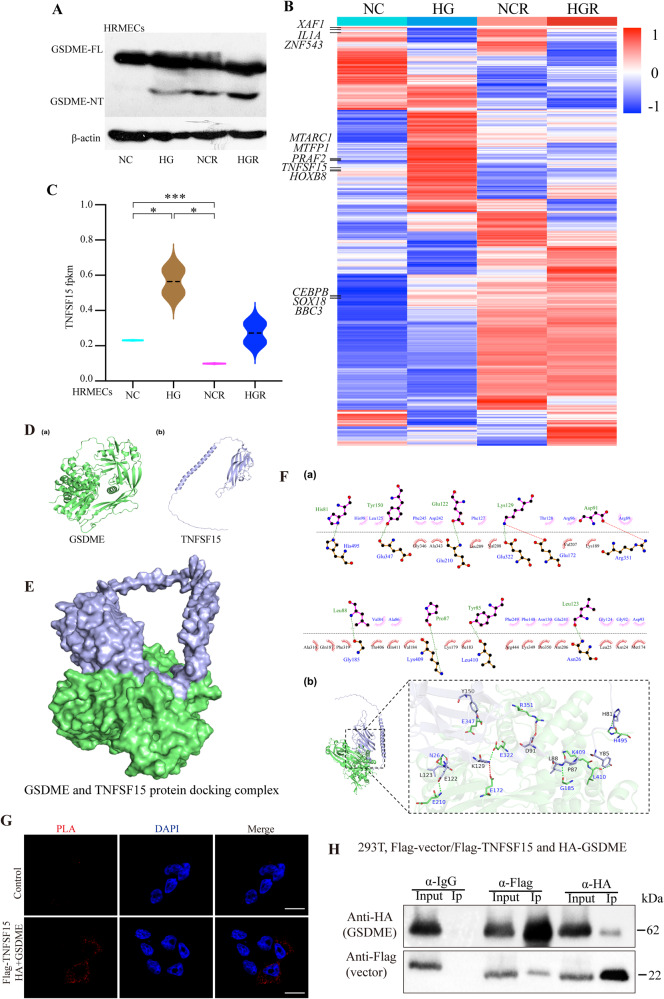
TNFSF15 inhibits pyroptosis by interacting with GSDME in DR. **A** WB assay of GSDME protein expression in HRMECs cells of NC, HG, NCR and HGR groups. **B** Heatmap showing upregulated and downregulated genes in RNA-seq data of HRMECs cells from NC, HG, NCR and HGR groups. **C** mRNA expression of TNFSF15 in RNA-seq data of HRMECs cells from NC, HG, NCR and HGR groups. *P < 0.01, ***P < 0.0001. **D** Three-dimensional structure of GSDME protein (a); Three-dimensional Structure of TNFSF15 Protein (b). **E** Structure of GSDME protein and TNFSF15 protein docking complex. **F** The interaction mode between GSDME protein and TNFSF15 protein (a: 2D interaction mode between proteins, toothed amino acids are hydrophobic interactions, and the green dotted line is hydrogen bonding; b: 3D interaction pattern between proteins). **G** In situ PLA assay detects the interaction between TNFSF15 and GSDME. Scale bars: 100 μm. **H** Co-IP experiment detects the interaction between exogenous TNFSF15 and GSDME in 293T cells. In the Co-IP experiment, we used Goat Anti-Mouse IgG HRP (Light Chain Specific) and Goat Anti-Rabbit IgG HRP (Heavy Chain Specific).

To screen for factors regulating GSDME-mediated pyroptosis during the pathogenesis of DR, we performed high-throughput transcriptome sequencing on HRMECs under four conditions: normal control (NC), high glucose (HG), normal control with Raptinal (NCR), and high glucose with Raptinal (HGR). Western blot validation showed that high glucose markedly promoted GSDME NT generation, which was further enhanced by Raptinal treatment in NC and HG cells (Fig. 4A). Further analysis of the RNA-seq differential genes revealed several genes significantly upregulated in HG vs NC, but repressed by Raptinal, including *MTARC1*, *MTFP1*, *PRAF2*, *TNFSF15*, *HOXB8*, etc (Fig. 4B). Notably, tumor necrosis factor superfamily member 15 (TNFSF15), an angiogenesis inhibitor that can slow DR progression by suppressing VEGF, drew our interest. Moreover, TNFSF15 was identified as an interacting partner of GSDME in our protein–protein interaction candidates screening (data were not shown). The results suggested that TNFSF15 may be interact with SDC3, NDUFS5, KCTD14, DDIT3, MYH7, RRH, ATXN10, SCAF1, STX8. Accordingly, RNA-seq data showed TNFSF15 was markedly upregulated in HG, but strongly inhibited by Raptinal, suggesting a protection in response to death in retinal cells (Fig. 4C).

**Fig. 5:**
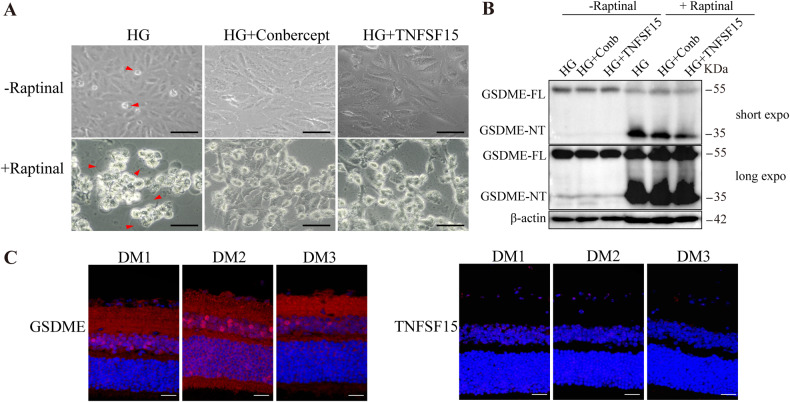
Recombinant TNFSF15 protein and Conbercept inhibits retinal cells pyroptosis. **A**, **B** ARPE-19 cells were treated with high glucose for 48 h, while TNFSF15 or 100 ng/ml conbercept were added to ARPE-19 cells, followed by Raptinal treatment. Cell pyroptosis phenotypes were observed (F). Scale bars, 50 μm. And the protein expression were determined by WB (G). **C** Immunofluorescence staining of TNFSF15 and GSMDE in retinal tissue of STZ-induced diabetic rats. Scale bars, 100 μm.

In order to study the binding region and interaction mode between GSDME protein and TNFSF15 protein molecules (Fig. 4D), the HDCOK program [25, 26] of professional protein–protein and protein-DNA /RNA docking was used for docking, and the docking score was calculated based on ITScorePP or ITScorePR iterative scoring function. The optimal Docking Score of GSDME protein and TNFSF15 protein was −264.76 kcal/mol (<−200), and the Confidence Score was 0.909 (>0.7). The results indicate that GSDME and TNFSF15 are highly likely to combine, and the composite model obtained by docking has high reliability (Fig. 4E). Subsequently, this study further analyzed the interaction pattern of spatial structure binding sites and binding regions between GSDME protein and TNFSF15 protein. Figure 5F shows the interaction model between GSDME protein and TNFSF15 protein. It can be seen from the figure that 8 groups of hydrogen bond interactions and 2 groups of salt bridge interactions are formed between GSDME protein and TNFSF15 protein. Specifically, among the hydrogen bonds formed by amino acids between two proteins, The GSDME proteins were Asn26, Glu172, Gly185, Glu210, Glu322, Glu347, Arg351, Lys409, Leu410 and His495. The amino acid residues from TNFSF15 protein are His81, Tyr85, Pro87, Leu88, Asp91, Glu122, Leu123, Lys129 and Tyr150. In addition, a large number of hydrophobic interactions between the two proteins promote further stable binding between the two proteins to form a complex (Fig. 4F). The interaction sites and modes between GSDME protein and TNFSF15 protein were studied by de novo modeling and molecular docking methods. The results showed that GSDME protein and TNFSF15 protein could form stable complex structures through hydrogen bonding and hydrophobic interaction, providing some guidance for the determination of the action site and the study of the action mode between two protein molecules. Subsequent in situ proximity ligation assay (PLA) and co-immunoprecipitation (Co-IP) verified the physical interaction between GSDME and TNFSF15 (Fig. 4G, H). Collectively, these results indicate that GSDME interacts with TNFSF15.

### TNFSF15 inhibit high glucose-induced pyroptosis via interacts with GSDME in retinal cells

We hypothesized that the interaction between TNFSF15 and GSDME might play an important role in retinal cell pyroptosis. Therefore, we then examined the regulatory mechanism between TNFSF15 and GSMDE in cells and diabetic rat models. ARPE-19 cells were treated with TNFSF15 recombined protein, and the anti-VEGF drug conbercept as a control. Surprisingly, the results demonstrated that both conbercept and TNFSF15 significantly suppressed the cleavage of GSDME and Raptinal-induced pyroptosis in the cells (Fig. 5A, B). Intravitreal injection of TNFSF15 AAV inhibited blood-retinal endothelial cell leakage and thus treated DR [23]. Therefore, in order to further verify the correlation between TNFSF15 and GSDME in vivo, we used IF to detect the expression of TNFSF15 and GSDME in retinal tissues of type 1 diabetic rats. Consistent with the results in vitro, TNFSF15 expression was significantly low in retinal tissue of diabetic rats, whereas GSDME was significantly overexpressed (Fig. 5C). Collectively, these data suggest TNFSF15 inhibits retinal cell pyroptosis and retards DR progression by interacting with GSDME.

## Discussion

Recent studies have implicated pyroptosis, an inflammatory form of programmed cell death, in the pathogenesis of DR. Our findings build on this growing body of research by showing that glucose-induced caspase-3 activation cleaves GSDME to trigger pyroptosis in retinal endothelial cells and RPE cells. More importantly, we identify TNFSF15 as a regulator of GSDME activity and an inhibitor of retinal cell pyroptosis, adding to our understanding of the molecular control of this process.

Multiple lines of evidence from our study as well as previous reports support targeting the TNFSF15-GSDME axis as a promising therapeutic strategy for DR. First, TNFSF15 expression increased under high glucose conditions, likely as an endogenous protective response, consistent with other research showing upregulation of TNFSF15 in diabetes [24]. Second, the pyroptosis inducer Raptinal downregulates endogenous TNFSF15 expression, releasing its inhibition on GSDME, further demonstrating the key role of this pathway. Third, exogenous TNFSF15 potently suppressed GSDME-dependent pyroptosis induced by high glucose and caspase-3 activation, highlighting its therapeutic potential [24]. Fourth, the anti-VEGF agent conbercept, which improves DR-associated neovascularization [17], also inhibited pyroptosis, suggesting pyroptosis blockade may broadly underlie efficacious DR therapies, as proposed by other studies [16, 27]. Finally, TNFSF15 exerts anti-pyroptotic effects by direct protein–protein interaction with GSDME, elucidating the mechanistic basis for TNFSF15’s protective actions.

Dual blockade of the TNFS15-GSDME pyroptosis axis along with VEGF inhibition thus provides a rational combination strategy by mediating complementary therapeutic mechanisms for more effective treatment of DR. TNFSF15 agonists could be developed to leverage the endogenous pyroptosis inhibitory effects of GSDME. Small molecule inhibitors directly suppressing GSDME [28]. Combined use of TNFSF15 agonists and GSDME inhibitors represents an ideal therapeutic regimen for DR. TNFSF15 agonists or GSDME inhibitors could each be combined with anti-VEGF agents, which may yield additive or synergistic effects. This is an active area of development, as anti-VEGF therapies like conbercept [17, 21] have demonstrated pyroptosis inhibition and DR improvement.

Future investigations should explore combination regimens leveraging TNFSF15-based pyroptosis inhibitors and anti-VEGF agents like conbercept. Testing synergistic treatment potentials in preclinical DR models is warranted. The domains in which TNFSF15 interacts with GSMDE will be further studied in subsequent projects. On the clinical translation side, pharmacological agents or biologics that boost endogenous TNFSF15 activity or directly disrupt the TNFSF15-GSDME interaction represent promising new additions to the DR therapeutic armamentarium.

In summary, our findings reveal TNFSF15 is a natural brake on GSDME-triggered retinal cell pyroptosis induced by diabetes. Targeting this newly identified cell death pathway and its regulatory mechanisms provides an exciting opportunity to develop novel treatments for this vision-threatening disease.

### Supplementary information

WB Raw Data

## Data Availability

Raw data for this article will be accessed on online database upon publication.
